# Immersive Virtual Reality (VR) when learning anatomy in midwifery education: A pre-post pilot study

**DOI:** 10.18332/ejm/191364

**Published:** 2024-08-28

**Authors:** Katrine Aasekjær, Bente Bjørnås, Halldis K. Skivenes, Eline S. Vik

**Affiliations:** 1 Faculty of Health and Social Sciences, Western Norway University of Applied Sciences, Bergen, Norway; 2 Haukeland University Hospital, Bergen, Norway

**Keywords:** anatomy, learning, pilot study, midwifery students, virtual reality, immersive

## Abstract

**INTRODUCTION:**

The integration of technology within teaching offers efficient and diverse learning opportunities. Studies have shown that the use of virtual reality (VR), improves anatomical knowledge and spatial understanding. The aim of this pilot study was to examine whether the utilization of immersive virtual reality goggles as a learning tool for anatomy increase midwifery students' knowledge, and to explore the potential of replacing traditional classroom teaching with VR.

**METHODS:**

We conducted a pre-post pilot study using a questionnaire before and after the use of VR as a learning tool in two cohorts of midwifery students in higher education. Cohort one had completed eight hours of classroom teaching of anatomy before participating in the VR session.

**RESULTS:**

The study included 44 midwifery students from two different classes at the same Master's program in midwifery at a university college in Norway. Student in both cohorts were in their first semester of midwifery studies and possessed a Bachelor's degree in nursing. Both cohorts had an increased average mean score in anatomical knowledge immediate after and 14 days after attending the learning session in VR. Students from the cohort that did not participate in anatomy lectures scored high on knowledge, both before and after the session in VR compared to the cohort that had additional classroom teaching in anatomy.

**CONCLUSIONS:**

Implementing VR as a learning tool, can contribute to increase spatial understanding and anatomical knowledge. By focusing on student learning in combination with learning activities and collaboration, the technology helps students gain understanding and knowledge.

## INTRODUCTION

Evidence-based healthcare education is important for ensuring quality care and patient safety^1^. Higher education has an important role in knowledge translation and strengthening healthcare students’ competencies and clinical skills^2^. Technology implementation in higher education has enhanced the possibilities of teaching complex concepts to students efficiently and with a wide variation and visualization^2,3^. An example of a complex subject is the teaching and learning of anatomy. Anatomy is an essential science in medicine and healthcare education, and anatomical knowledge is important for developing skills and becoming competent practitioners^4-7^. Students from medicine and nursing have claimed that constructing knowledge about anatomical structures and how various bones, muscles, nerves, and other structures are located and relate to one another is difficult or challenging^3^. Understanding and perceiving spatial dimensions and how human structures relate to one another is difficult to learn using two-dimensional resources, while anatomical structures are three-dimensional^6^. A review comprising eight quantitative studies found that the use of three-dimensional learning methods instead of two-dimensional methods showed increased anatomical and spatial anatomy knowledge among students^6^.

A known and acknowledged learning resource for exploring and understanding anatomy is the use of virtual reality (VR) goggles. Using VR, the students can immerse in a synthetic anatomical virtual environment, where they can move around and interact, and the students’ learning is influenced by stimuli actions within the virtual environment^8^. Several systematic reviews state that VR can enhance motivation for learning and preserve knowledge and in-depth learning among students in healthcare^9-13^. Few studies have demonstrated the enhancement of learning outcomes in anatomy using VR in midwifery education. Therefore, we conducted a pre-post pilot study to investigate whether VR could affect the learning and knowledge of pelvic anatomy among midwifery students at a university college in Norway. The pre-post pilot study aimed to examine whether the utilization of immersive virtual reality goggles as a learning tool for anatomy increases midwifery students’ knowledge and to explore the potential of replacing traditional classroom learning methods with VR technology.

## METHODS

We conducted a pre-post pilot study investigating midwifery students’ pelvic anatomy knowledge before and after using VR technology as a learning tool. A pilot study is considered applicable when evaluating or improving education, which is the aim of the study^14,15^.

### Intervention – VR technology learning tool

**Figure 1:**
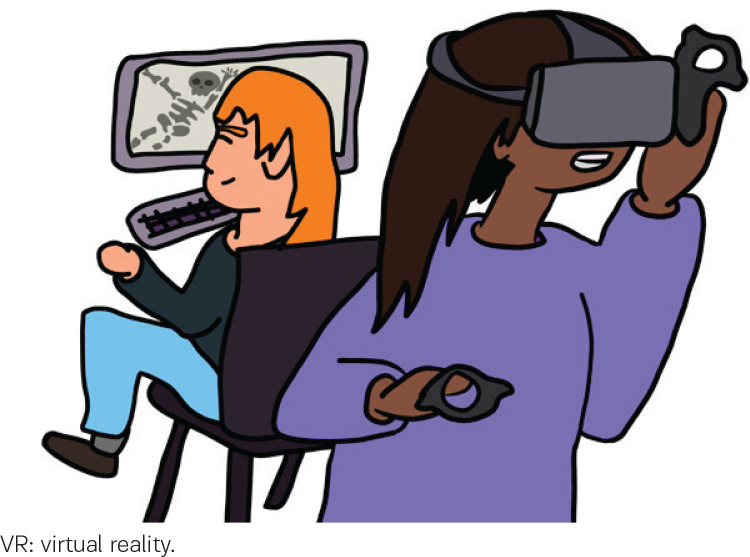
Illustrating a person wearing headmounted display (HMD) and hand-controllers in a virtual environment (with permission from Linus Andresson Vik)

Using head-mounted display (HMD) and hand-held controllers, the students were introduced to the female pelvic anatomy using the 3D Organon VR platform, a medical and healthcare platform for teaching and learning anatomy^16^. The source of the 3D model in this study was the 3D Organon female reproductive organ. The model includes all the female pelvic organs, muscles, ligaments, bones, nerves, and blood vessels in a non-pregnant woman (Figure 1). When the students entered the virtual environment, they could rotate the female pelvis into different positions, zoom in and out, and move structures to understand the spatial dimensions. Also, when hovering the hand-held controllers over the 3D model, a label is displayed to identify the present structure.

All the students participated in a two-hour session using 3D immersive VR to learn the female pelvic anatomy (Figure 1). The students collaborated in groups of two, with a teacher as a facilitator and discussion partner during the two-hour session. The students were given a task to discuss the three stages of labor and identify and explain the different anatomical structures to one another. In addition, because the VR platform did not contain a fetus, the students had to demonstrate to their peers how the fetus rotated through the female pelvis, using their head and body in the virtual environment.

### Sample and data collection

First-year midwifery students from two different cohorts from the same Master’s program in midwifery were invited to participate in the pilot study. Each cohort consisted of 25 midwifery students, with a mean age of 27 years in both cohorts. Most students from both cohorts had one year of experience as a nurse before enrolling in the Master’s program in midwifery (Admission Office 2021, Western University of Applied Science). None of the students had experience using VR prior to the anatomy session, but the students from cohort 1 had all participated in two traditional lectures in anatomy before entering the VR sessions in anatomy. Each lecture spanned four hours, amounting to a total of eight hours of lecture time. From a teaching point of view, this was the only distinguishing factor between the two cohorts. Students from both cohorts were given a one-hour tutorial on how to view and interact in the virtual environment prior to the anatomy session in VR.

A total of 50 students were eligible for the study: 25 midwifery students from each cohort were recruited before the anatomy session in the VR laboratory on campus. All participants provided written consent, and we collected the data in September 2019 (cohort 1) and September 2020 (cohort 2). One student from each cohort was excluded from the study because they withdrew from post-tests I and II.

Data were collected using a self-reporting questionnaire. The questionnaire contained 11 questions concerning knowledge of spatial understanding, bones, muscles, and nerves. Each test had a maximum possible score of 18 correct answers. The data were collected in a classroom under the observation of a teacher. We collected data before, immediately after, and 14 days after the students participated in the VR anatomy session. In addition to assessing the student’s knowledge, the questionnaire delivered 14 days after the VR intervention also contained an open-ended question asking the students about their experience using VR as a learning tool.

### Ethics

The Norwegian Social Science Data Service (Sikt) no 99-6813 assessed the study. The data were plotted by a person who had no recognition of the students nor an engagement in the Master’s program in midwifery. All participants were given written and oral information about the study.

### Analysis

The difference between the students’ scores (i.e. the number of correct answers) for the pre-test and post-test determined the knowledge gained after exposure to the VR tool. Descriptive statistics, comprising frequencies and percentages, were employed to present the results. To assess learning achievement before and after anatomy instruction in the virtual environment, both independent and paired sample t-tests were used, with the outcomes presented as p-values. Additionally, independent and paired t-tests were conducted to investigate performance differences between the two cohorts. The significance level for all tests was set at 0.05. Data were analyzed using the Statistical Package of the Social Sciences (IBM SPSS version 27).

## RESULTS

**Figure 2:**
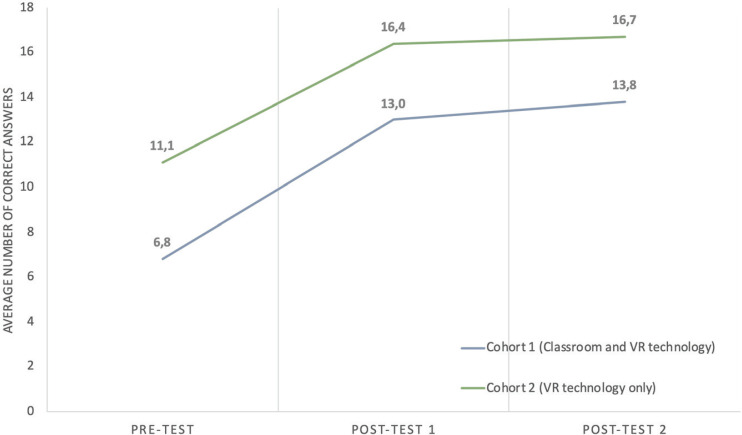
Average number of correct answers in pelvic anatomy knowledge for pre-test, post-test I, and posttest II for cohort 1 (2019) and cohort 2 (2020)

A total of 48 students, 24 assigned in each cohort, answered the questionnaires measuring the students’ knowledge before (pre-test), immediately after (post-test I), and 14 days after (post-test II) the anatomy lecture in VR. The knowledge of the female pelvis increased for both cohorts after the intervention using virtual reality goggles as a learning instrument when learning female pelvic anatomy. This knowledge achievement continued to increase when we tested the students’ knowledge 14 days after the intervention using VR goggles (Figure 2).

Our results show an increase in the number of correct answers from pre-test to post-test I (p<0.001) and from post-test I to post-test II (p<0.001) for both cohorts (Figure 2). Compared to the cohort solely exposed to virtual reality (VR), the cohort who received both traditional lectures and VR exhibited a lower number of correct answers during the pre-test (p<0.001).

The performance of students in both cohorts across the three distinct tests is outlined in Table 1. The table shows that the cohort exclusively exposed to virtual reality (VR) performed proficiently in three out of four domains: anatomical navigation, bones, and muscles. Conversely, the cohort receiving traditional lectures and VR excelled notably in the smallest domain: nerves.

**Table 1:** Knowledge of anatomical navigation, bones, muscles, and nerves for pre-test, post-test I, and post-test II, for cohort 1 and cohort 2, demonstrated in the number of correct answers among midwifery students at a university college from 2019 to 2020, Norway (N=50)

	*Cohort 1 Correct Answers (N=24)*			*Cohort 2 Correct Answers (N=24)*		
	*Pre-test n (%)*	*Post-test I n (%)*	*Post-test II n (%)*	*Pre-test n (%)*	*Post-test I n (%)*	*Post-test II n (%)*
**Anatomical navigation**						
What does the term superior mean?	20 (83.3)	24 (100)	24 (100)	24 (100)	24 (100)	24 (100)
What does the term anterior mean?	17 (70.8)	20 (83.3)	23 (95.8)	24 (100)	24 (100)	24 (100)
What does the term superficial mean?	12 (50.0)	22 (91.6)	20 (83.3)	14 (58.3)	23 (95.8)	24 (100)
The promontory is an important landmark in the pelvic inlet. How is the promontory positioned in relation to the symphysis?	8 (33.3)	20 (83.3)	24 (100)	16 (66.7)	20 (83.3)	24 (100)
Is levator ani positioned superior or inferior to the urogenital diaphragm/triangle?	7 (29.2)	18 (75.0)	20 (83.3)	16 (81.6)	20 (83.3)	19 (79.2)
Average correct answers in anatomical navigation	46 (53.3)	104 (86.6)	111 (92.5)	98 (81.6)	114 (95.0)	115 (95.8)
**Bones**						
Which bones does the pelvic consist of? *	31 (43.3)	29 (40.3)	41 (56.9)	59 (81.9)	66 (91.6)	65 (90.3)
Os pelvic is a fusion of three bones. What are the names of these bones? *	30 (41.7)	64 (88.9)	67 (93.0)	43 (59.7)	43 (59.7)	68 (94.4)
Iliaca crest ends in two distinct structures – what are they called? **	5 (10.4)	18 (37.5)	16 (33.3)	10 (20.8)	32 (66.7)	28 (58.3)
Average correct answers in bones	65 (34.4)	111 (57.8)	124 (64.6)	112 (58.3)	161 (83.9)	161 (83.9)
**Muscles**						
Which muscles does levator ani consist of?	6 (8.3)	42 (58.3)	35 (48.6)	36 (48.6)	59 (81.9)	61 (91.7)
Which muscles are included in the urogenital diaphragm/triangle? *	9 (12.5)	34 (47.2)	37 (51.4)	10 (13.9)	37 (51.4)	41 (56.9)
Average correct answers in muscles	15 (70.8)	76 (52.7)	72 (50.0)	6 (31.9)	96 (66.7)	102 (70.8)
**Nerves**						
The ischial spine is an important landmark in the pelvis. Which nerve passes medial to the ischial spine?	17 (70.8)	22 (91.7)	23 (95.8)	10 (41.7)	22 (91.7)	22 (91.7)

* Explain …

** Explain …

A total of 91.6% (n=44) of participants responded to a final open-ended question, which inquired about their experience with using VR. Regardless of the cohort, all their comments on using VR as a learning tool for gaining knowledge in anatomy were positive. The students reported that VR facilitated understanding the complexity of the female pelvis in relation to the fetus, enhanced the visualization of the pelvic details, eased the navigation, and clarified anatomical relations. Both cohorts experienced an increased understanding of the spatial relations between the different structures when using VR as a learning tool. They also considered that small group activities were more helpful than working alone due to the complexity of the subject matter.

## DISCUSSION

A pre-post pilot study investigated whether VR improved the student’s anatomical knowledge. The pilot study showed an increased knowledge achievement in all the students participating in the study, both immediately and 14 days after the lecture in anatomy. The mean knowledge score was highest in the cohort that only received VR as a learning tool (cohort 2). The knowledge score in anatomy in this cohort was higher in both pre- and post-VR sessions. We observed sustainability in knowledge 14 days after the lecture in anatomy (post-test II), and this sustainability was observed in both cohorts. Our findings indicate the potential of implementing immersive VR in midwifery education to understand the female pelvis and cardinal movements of the fetus.

Traditionally, anatomy is taught through classroom lecturing, and within midwifery education in Norway, dissection of cadavers has not been an available learning tool. The midwifery students in our study demonstrated increased knowledge using VR as a learning tool when learning anatomy. It is argued that anatomy is an essential science within healthcare education and important knowledge in becoming a competent practitioner^4-7^. The use of technology, such as VR, can enhance interest among students and provide them with better conditions to understand complex information and phenomena, such as anatomy. In our study, the midwifery students increased and maintained their knowledge of anatomy after using VR technology. This maintenance suggests that VR stimulated the students to reflect and perceive the knowledge experienced in the VR environment. Knowledge develops and constructs in collaboration and interaction with others^17^, and irrespective of which teaching method is used, interaction and direct experience are considered effective teaching strategies^4,18^. The students who only received teaching through VR technology had a higher average mean score in knowledge (pre-test) before the VR session. This result could indicate that the students were forced to take a more active approach to their own learning by removing the classroom teaching. Lifelong learning and in-depth knowledge are goals in higher education, and taking responsibility for their learning is a prerequisite for in-depth learning^1,17,18^. The fact that VR is a more active and collaborative teaching method could explain why both cohorts acquired a higher level of knowledge after attending their session in VR.

We observed an increased knowledge of spatial understanding among the midwifery students. Many healthcare students find it difficult to understand how anatomical structures relate to one another^5^. The increased spatial knowledge among the midwifery students in our study could be the impact of the VR environment that facilitates the opportunity to create a realistic learning environment and that this environment made it easier to identify, visualize, and understand how the different anatomical structures relate to one another. Identifying three-dimensional anatomical structures using twodimensional learning tools such as books and lectures is reported in other studies as difficult when visualizing and conceptualizing anatomical structures^5,6,19^. Actual anatomical knowledge and spatial anatomy knowledge have been shown to increase using three-dimensional methods instead of two-dimensional^13^.

Using VR as a learning tool, the midwifery students reported that VR facilitated the visualization and understanding of the complex interaction between the female pelvis and the fetus. The task in the VR session was actual labor. Combining clinical examples with anatomy subjects might increase the midwifery students’ understanding of why knowledge about anatomy is important in becoming a competent practitioner. Studies have shown that combining relevant clinical examples with complex subjects, increases knowledge and understanding, in addition to enhancing student awareness of why the subject is relevant to learning^20,21^.

The use of digital tools in teaching provides a variation in teaching and creates learning arenas that are not possible to create in conventional teaching of anatomy. By being able to take the fetus position, the midwifery students had to simulate the rotation of the fetus by using their head and body through the female pelvis. Higher education plays an important role in knowledge translation, strengthening students’ competencies and clinical skills. To prepare and enable students to make the right decisions at the right time, healthcare education must offer a wide range of realistic and authentic learning opportunities. Studies have reported that using authentic learning modes creates meaningfulness and motivation when studying anatomy^21,22^. In addition to variation, VR and digital technology could prepare students for their professional occupations; thus, society is increasingly implementing and adopting new technology.

By implementing VR, the students in our study had to be more active and collaborate with peers. Using immersive three-dimensional models in VR allowed midwifery students to interact with the environment, participate directly, and learn by doing. This active approach altered the position of the students from a passive recipient to an active participant in their learning. The midwifery students examined the anatomical structures and organs from different angles, zooming in and out and discussing the different anatomical layers and positions with their peers. VR transformed the teaching into a more learner-centered approach, and by doing so, the teaching also became collaborative and student-active. Our experience aligns with other studies demonstrating that VR can facilitate students’ motivation and engagement and enhance critical thinking by becoming collaborative and interactive^21,23,24^.

### Strengths and limitations

In our study, the students experienced increased knowledge of using VR as a learning tool. Whether this increase is associated with VR or could be explained with a more active and collaborative approach in the presentation of anatomy to the students was beyond the scope of this study. Using VR, the students are invited into an interactive and collaborative environment, so it is likely that VR significantly impacted the student’s ability to understand and learn anatomy. Future research should compare VR to other active learning strategies. We conducted a pre-post pilot study to measure change in knowledge among midwifery students. By measuring the baseline knowledge before the VR session, we could compare this with the knowledge gained after the session. Then, we could decide whether the intervention (VR) had an impact on students’ knowledge or not. We compared two cohorts who both received the same intervention. Our findings would have been strengthened if one of the cohorts had not received VR as a learning tool. Hence, this pilot study demonstrated that the teaching method increased the knowledge of all students participating in the study. In the future, larger studies with a randomized design should be conducted. This study is limited in size, and selection bias may account for the observed differences between the two cohorts. Alternatively, variations could stem from factors such as individual motivation for learning anatomy, influenced by whether students receive traditional lectures. Larger studies employing diverse methodologies are necessary to gain a clearer understanding of these differences.

## CONCLUSIONS

Implementing VR as a learning tool can increase spatial understanding and anatomical knowledge. By focusing on student learning in combination with collaboration and student active learning, the technology helps students gain understanding and knowledge. In addition, VR and digital technology are described as fun and could increase the student’s motivation for further learning.

## Data Availability

The data supporting this research are available from the authors on reasonable request.
